# Significance of Signal Averaged Electrocardiography in Patients with Advanced Heart Failure and Intraventricular Conduction Delay

**Published:** 2010-05-05

**Authors:** Mohammad Alasti, Majid Haghjoo, Abolfath Alizadeh, Mohammad Hossein Nikoo, Hamid Reza Bonakdar, Bita Omidvar, Ali Kharazi, Bahman Cheraghian

**Affiliations:** 1 Department of Cardiology, Imam Khomeini Hospital, Jondishapour University of Medical Sciences, Ahwaz, Iran; 2 Department of Pacemaker and Electrophysiology, Rajaie Cardiovascular Medical and Research Center, Iran University of Medical Sciences, Tehran, Iran; 3 Department of Electrophysiology, Kosar Hospital, Shiraz, Iran; 4 Department of Cardiology, Heshmat Hospital, Gilan University of Medical Sciences, Rasht, Iran; 5 Department of Internal Medicine, Golestan Hospital, Jondishapour University of Medical Sciences, Ahwaz, Iran; 6 Department of Electrophysiology, Bahman General Hospital, Tehran, Iran; 7 Department of epidemiology, Faculty of nursing, Abadan, Iran

**Keywords:** heart failure, tissue Doppler echocardiography, signal averaged electrocardiography, left ventricular dyssynchrony

## Abstract

**Aims:**

Signal averaged electrocardiography is a noninvasive method to evaluate the presence of the potentials that are generated by tissues, activated later than their usual timing in the cardiac cycle. The purpose of this study was to demonstrate the correlation of data obtained via signal averaged electrocardiography and left ventricular dyssynchrony.

**Methods:**

We included the patients with advanced systolic left ventricular dysfunction (ejection fraction ≤ 35%) and intraventricular conduction delay. All patients underwent surface 12-lead electrocardiography, signal averaged ECG, and tissue Doppler echocardiography.

**Results:**

The study included 72 patients with mean age of 56.45±13.59 years. Mean QRS duration was 0.14 ± 0.02 sec; 63.9% of patients had left bundle branch block. Linear regression demonstrated significant correlations between filtered QRS duration and interventricular mechanical delay (P<0.000, Y= 0.41X-24.76), root mean square 40 and peak velocity difference (P: 0.001, Y=-0.39X+109.72), root mean square 40 and Ts-SD-12 (P:0.026, Y=-o.26X+40.08), low amplitude signals duration and peak velocity difference (P<0.000, Y=0.44X+67.3) and finally low amplitude signals duration and Ts-SD-12 (P:0.31, Y=0.26X+28.23) as well. Area under the curve in ROC of filtered QRS duration was significant for the detection of interventricular mechanical delay. Areas under the curves in ROC of low amplitude signal duration and root mean square 40 were significant for the detection of peak velocity difference.

**Conclusions:**

Signal averaged electrocardiography can have a role in predicting the amount of ventricular dyssynchrony. The duration of low amplitude signals and root mean square 40 have significant linear relations to some indices of intraventricular dyssynchrony.

## Introduction

Cardiac resynchronization therapy (CRT) is an effective treatment for patients with severe heart failure and concomitant wide QRS complex on ECG. It can improve cardiac function, clinical status, quality of life and even survival in the above mentioned group [1]. Current selection criteria for suitable candidates for CRT are as follows: moderate to severe heart failure (NYHA class III or IV) despite optimal medical therapy, systolic dysfunction (left ventricular ejection fraction (LVEF) ≤ 35%) and a wide QRS complex (QRS duration ≥120 ms) [2]. Recent studies have demonstrated that assessment of inter and particularly intraventricular dyssynchrony may lead to identification of potential responders to CRT [3]. Commonly, echocardiography and tissue Doppler imaging (TDI) are being used to provide a complete description of mechanical abnormalities due to dyssynchrony. 

High resolution ECG is designed for body surface recording of the cardiac signals that are not visible on standard ECG. Signal averaging is an approach to produce high resolution ECG. In this type of electrocardiography, late potentials are generated by tissues, activated later than their usual timing in the cardiac cycle [4]. Meanwhile, it is expected to have late potentials in patients with intraventricular conduction defects. We, therefore, designed an observational study aimed at evaluating the possible correlation between data obtained by signal averaged electrocardiography (SAECG) and dyssynchrony indices in echocardiography.

## Methods

### Study Population

The patients included in the study were selected consecutively among those referred to our centers with impression of heart failure and subsequent treatment. Following inclusion criteria were considered: (1) advanced systolic left ventricular dysfunction (left ventricular ejection fraction ≤ 35%); (2) underlying cause being idiopathic dilated cardiomyopathy (DCM) or  ischemic heart disease (IHD), (3) NYHA class III or IV, (4) QRS complex duration ≥ 120 ms. All patients signed written informed consent. We excluded patients with: (1) non-sinus rhythm; (2) previous pacemaker implantation; (3) a recent myocardial infarction (< 3 months); (4) severe aortic disease (5) right bundle branch block. All patients underwent a standard 12-lead ECG, a SAECG and an echocardiographic examination, including specific evaluation of interventricular and intraventricular dyssynchrony. The study was approved by the institutional review board.

### Echocardiography

Imaging was done in the left lateral decubitus position, recording  parasternal and apical views (standard long-axis , two- and four- chamber images) using a commercially available system (Vingmed 7, General Electric, Milwaukee, WI, USA). A 3.5-MHz transducer was used. Left ventricular volumes (end-systolic, end-diastolic) and left ventricular ejection fraction were calculated from the conventional apical two- and four-chamber images, by the biplane Simpson's technique. Mitral regurgitation was graded according to the jet area method.

The aortic and pulmonary pre-ejection times were measured, starting from the beginning of QRS complex to the beginning of the flow velocity curve. The difference between the two values determined interventricular mechanical delay (IVMD), as the index of interventricular dyssynchrony. M-mode was used to obtain septal to posterior wall motion delay (SPWMD). Color-coded tissue Doppler imaging was acquired in 2-D mode from the apical 4-chamber and 2-chamber views to assess myocardial regional function. Off-line analysis of the time to peak velocity was performed at the level of the basal and middle segments of the interventricular septum and the lateral, inferior, anterior, posterior and anteroseptal walls, with reference to the QRS complex. The peak velocity difference (PVD) was measured as the time difference between the earliest and latest contracting segment. To determine septal to lateral wall delay (SLWD), the sample volume was placed in the basal portion of the septum and the lateral wall. Peak systolic velocities and time-to-peak systolic velocities were obtained and septal-to-lateral delay in peak velocity was calculated. In addition to PVD, SLWD and SPWMD, the standard deviation of time to peak systolic velocity of the 12 LV segments (Ts-SD-12) and six basal segments (Ts-SD-6) were calculated in each patient as indices of intraventricular dyssynchrony.

### Electrocardiography

QRS duration was measured on surface ECG. ECG was recorded at a speed of 25 mm/second and a scale of 10 mm/mV. QRS duration was measured as the widest QRS complex in precordial leads. QRS durations equal or more than 0.12 sec, no q-wave but slurred, broad R waves in leads I, aVL and V6 and rS or QS deflections in lead V1 were considered as the ECG features of left bundle branch block (LBBB). On the other hand, QRS durations equal or more than 120 ms, broad and notched R waves in leads V1 and V2 and deep S deflections in left precordial leads and I were noted as the ECG features of right bundle branch block (RBBB). A prolonged QRS not associated with the typical features of bundle branch block, was labeled as nonspecific intraventricular conduction delay (IVCD).

### Signal averaged electrocardiogram

The filtered QRS duration, root-mean-square voltage of the terminal 40 milliseconds in the filtered QRS complex (RMS40), and duration of low-amplitude signals <40 μV in the terminal filtered QRS complex (LAS40) were calculated, using signal averaged ECG (Hellige EK 56, Marquette, Freiburg, Germany) with noise level <0.3 μV and high-pass filtering of 40 Hz. Two hundred cardiac cycles were acquired and averaged each time. The key hardware elements of the system were an amplifier, a convertor for digitization of signals, a signal processor and a printer. In this system, a computer algorithm was utilized to identify QRS onset and offset. Filtering was applied to reduce the residual noise and improve identification of low potentials.

### Statistical analysis

Continuous data were expressed as mean ± standard deviation values. Linear regression analysis was the chosen method for evaluating the association between signal averaged data and echocardiographic characteristics of patients with dyssynchrony indices. A P value less than 0.05 was considered to be statistically significant.

Receivers operating characteristic curves (ROC curves) were used to determine the area under the curve (AUC) and the optimum cut off level of each SAECG indices and the 95% confidence intervals (CI) were obtained. The optimum value is the value that minimizes the distance from the upper left corner of the plot frame.

## Results

The study population consisted of 72 patients; 55 (76.4%) men and 17 (23.6%) women. The mean age was 56.45±13.59 years. The underlying etiology of heart failure was ischemic in 63.9% of patients. Seventy one (98.6%) patients were in NYHA class III. The baseline characteristics of the study population are summarized in Table 1.

### Electrocardiographic findings

All patients had sinus rhythm on ECG. Mean QRS duration was 0.14 ± 0.02 sec (range: 0.12–0.2 sec); 46 (63.9%) patients had LBBB morphology and 26 (36.1%) patients had nonspecific IVCD morphology. The mean filtered QRS duration was 153.81 ± 20.61 ms (range= 116-234 ms). The mean RMS40 was 14.36± 13.43 μV (range=3-73 μV) and the mean LAS40 was 46.85± 22.36 ms (range=4-98 ms).

### Echocardiographic findings

Considering echocardiography findings, the mean left ventricular ejection fraction (LVEF) was 18.14 ± 5.67% (range 10-33%). 15.3% of patients had severe mitral regurgitation (MR). Detailed echocardiographic characteristics of the patients are presented in Table 1.

### Correlation of SAECG findings and echo indices for mechanical dyssynchrony

**Figure 1:**
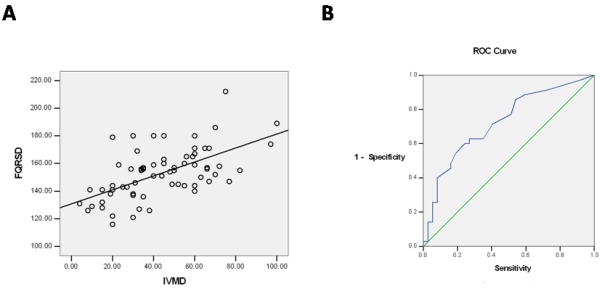
A- Relationship between the interventricular mechanical delay (IVMD, ms) and filtered QRS duration (fQRS, ms) (r = 0.41, P < 0.000). B- ROC curve for filtered QRS duration to detect interventricular mechanical delay.

Linear regression demonstrated a significant correlation between filtered QRS duration and IVMD (P<0.000, Y= 0.41X-24.76) (Figure 1A), whereas there was no significant correlation between filtered QRS duration and echo indices of intraventricular dyssynchrony (Table 2).

**Figure 2:**
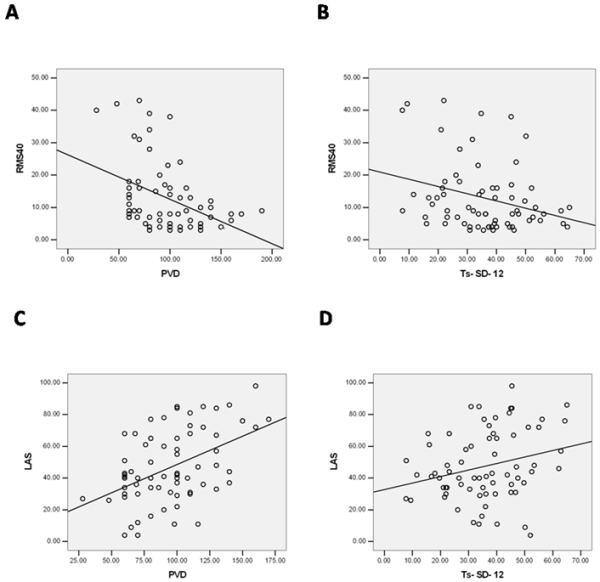
A- Relationship between the peak velocity difference (PVD, ms) and root mean square 40 (RMS40, ms) (r=-0.39, P=0.001). B- Relationship between the standard deviation of time to peak systolic velocity of the 12 LV segments (Ts-SD-12, ms) and root mean square 40 (RMS40, ms) (r=-0.26, P=0.026). C: Relationship between peak velocity difference (PVD, ms) and low amplitude signals duration (LAS, ms) (r=0.44, P<0.000). D: Relationship between the standard deviation of time to peak systolic velocity of the 12 LV segments (Ts-SD-12, ms) and low amplitude signals duration (LAS, ms) (r=0.26, P=0.031).

There were significant relations between RMS40 and PVD (P=0.001,Y=-0.39X+109.72) (Figure 2A) and RMS40 and Ts-SD-12 (P=0.026, Y=-o.26X+40.08) (Figure 2B). There was no statistically significant relation between RMS40 and other echo indices (Table 3).

 A significant correlation was found between low amplitude signals duration and PVD (P<0.000, Y=0.44X+67.3) (Figure 2C) and between low amplitude signals duration and Ts-SD-12 (P=0.031, Y=0.26X+28.23) (Figure 2D). Linear regression demonstrated no relation between LAS duration and other echo indices (Table 4).

### ROC Curves

According to previous studies, IVMD of 40 ms was considered as significant interventricular dyssynchrony and PVD of 110 ms and Ts-SD-12 of 33 ms were considered as cut off values of significant intraventricular dyssynchrony [3].

Area under the curve in ROC of filtered QRS duration for detection of interventricular dyssynchrony was 0.791 (%95 CI, 0.665-0.918, P=0.000) (Figure 1B). The curve indicated that sensitivity of 85% and specificity of 76% were obtained with a filtered QRS duration of 144 ms as cut off value to distinguish patients with interventricular dyssynchrony.

**Figure 3:**
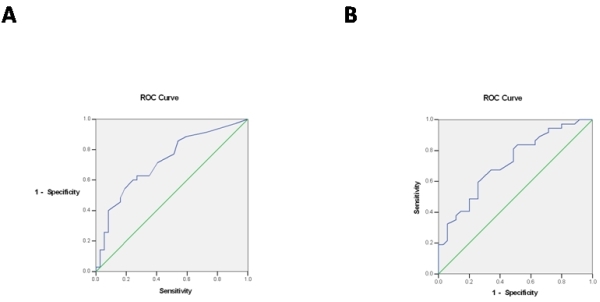
A- ROC curve for root mean square 40 to detect peak velocity difference as an index of intraventricular dyssynchrony. B: ROC curve for low amplitude signals duration to detect peak velocity difference as an index of intraventricular dyssynchrony.

Area under the curve in ROC of RMS40 for the detection of PVD was 0.724 (%95 CI, 0.606-0.842, P=0.001) (Figure 3A). Specificity and sensitivity were 63% and 65%, with a cut off value of 9.5 μV, and 91% and 21% with a cut off value of 4.5 μV. Presence of RMS40 of 20 μV had 92% sensitivity and 31% specificity for detection of significant PVD.

Area under the curve in ROC of LAS for the detection of PVD was 0.720 (%95 CI, 0.604-0.837, P=0.001) (Figure 3B). Specificity and sensitivity were 49% and 84%, with a cut off value of 35 ms. Presence of LAS of 39 ms had 52% sensitivity and 73% specificity and presence of LAS of 70 ms had 68% sensitivity and 94% specificity for detection of significant PVD.

SAECG indices did not show any significant AUC for detection of other echo parameters.

## Discussion

Currently, CRT is considered an important therapy for patients with advanced heart failure and concomitant wide QRS complex. Prolongation of QRS (>120 ms) occurs in 14% to 47% of patients with heart failure. LBBB occurs more commonly than RBBB (25% to 36% vs. 4% to 6%) [5]. Prolongation of QRS is a significant predictor for left ventricular systolic dysfunction in patients with heart failure. One heart failure study indicated that the incidence of QRS prolongation increased from 10% to 32% and 53% when patients moved from New York Heart Association (NYHA) functional class I to class II and III, respectively [5].

However, QRS duration measurement is not an optimum way to select suitable candidates for CRT. Analysis of the CRT patients have shown that despite prolonged QRS duration, at least 30% of the patients did not respond to CRT and there were no significant difference in baseline QRS duration, between clinical and echocardiographic CRT responders versus non-responders [6]. Kashani et al. evaluated the value of baseline QRS duration by analyzing 34 CRT studies including 2,063 patients. The authors concluded that despite a significant reduction in QRS after CRT initiation in 32 studies, difference in baseline QRS duration between clinical responders to CRT versus non-responders, was only reported in one study (190 ± 30 ms versus 171± 21 ms, P < 0.01) [5]. Response to CRT has been related to the presence of cardiac dyssynchrony prior to implantation. So the combination of traditional selection criteria, complemented by demonstration of left ventricular dyssynchrony, may allow more accurate prediction of response to CRT [7]. Several techniques have been proposed to quantify cardiac dyssynchrony in heart failure patients, i.e., echocardiography techniques, magnetic resonance imaging, nuclear imaging, computed tomography and body surface potentials [3]. Echocardiography is currently used as the most practical technique to assess ventricular dyssynchrony candidate for CRT implantation. However, being time consuming is its limiting disadvantage.

The signal averaged electrocardiography is a non-invasive test for risk stratification of sudden cardiac death, especially in survivors of MI. This technique results in an improvement of the signal to noise ratio, thus allowing analysis of signals that are too small to be detected by routine measurement [8]. Our data showed that there was a significant linear relation between low amplitude signals duration and PVD and Ts-SD-12, although the correlation coefficient was not good. According to previous studies, a PVD of ≥110 ms at baseline is predictive of LV reverse remodeling at the 3-month follow-up (sensitivity 97%, specificity 55%). Also, Ts-SD-12 with a cut-off value of ≥33 ms is a good index to predict response to CRT (sensitivity: 90%, specificity 83 %) [3]. Our study showed that we could predict the amount of dyssynchrony with respect to the duration of low amplitude signals and RMS40. So, the presence of low amplitude signals duration  ≥ 70 ms in signal averaged electrocardiography implies the presence of significant intraventricular dyssynchrony (specificity: 94%) and the presence of RMS40 ≤ 4.5 μV implies the presence of significant intraventricular dyssynchrony (specificity: 91%).

Like QRS duration in surface ECG, the amount of filtered QRS duration had only a significant relation to interventricular dyssynchrony. The presence of fQRS of 144 ms indicates the presence of significant interventricular dyssynchrony with 76% specificity. According to previous studies, intraventricular dyssynchrony is more important for prediction of CRT response than interventricular dyssynchrony [3]. So filtered QRS duration, like QRS duration in surface ECG, has a limited value.

Our results are in contrast with the results of two recent studies [10,11] which revealed a significant relationship between fQRS and inter and intraventricular dyssynchrony but no relationship between LAS duration and RMS40 and ventricular dyssynchrony. So, further studies with greater number of patients are needed to answer the questions and verify the clinical significance.

## Study limitations

Several limitations of the study also need to be acknowledged. First, this is a small study and evaluation of a large group of patients is needed to confirm our results. Second, we used echocardiography to assess ventricular dyssynchrony as the standard. According to previous studies, most echocardiographic techniques have limited interobserver reproducibility and significant training is required [3]. Third, we evaluated the patients with heart failure and QRS duration equal or more than 120 msec. Ghioa et al demonstrated that there are a substantial proportion of heart failure patients with normal QRS duration who may also exhibit dyssynchrony [9]. Therefore, a study for evaluation of patients with heart failure and normal QRS duration should be designed. Fourth, our patients were a heterogeneous group. Patients with ischemic or idiopathic dilated cardiomyopathies and patients with LBBB or nonspecific IVCD all were included in this study.

## Conclusions

According to our data, signal averaged electrocardiography can have a role in predicting the amount of inter- and intraventricular dyssynchrony. The duration of low amplitude signals and root mean square 40 have significant linear relations to some indices of intraventricular dyssynchrony (PVD and Ts-SD-12). The clinical significance of our findings warrants further investigation.

## Figures and Tables

**Table 1:**
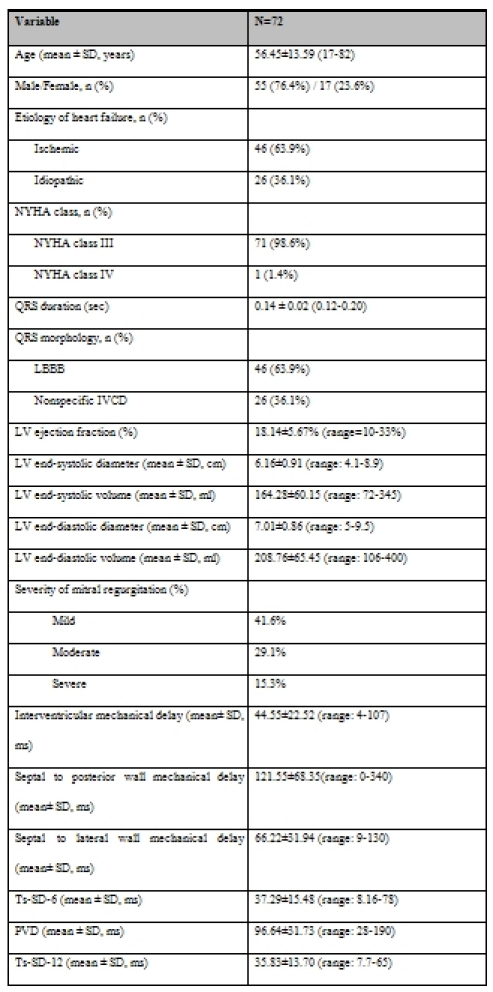
Baseline clinical and echocardiographic characteristics of study population

**Table 2:**
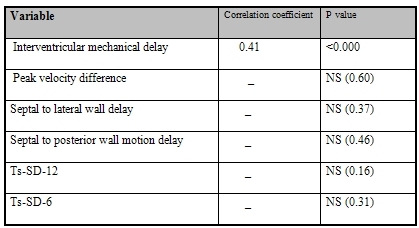
Correlation of filtered QRS duration and echo indices for mechanical dyssynchrony

**Table 3:**
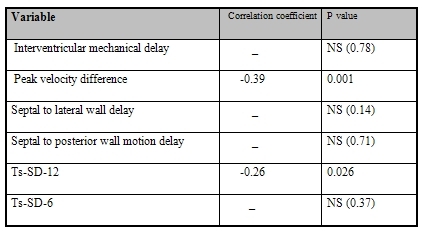
Correlation of root mean square 40 and echo indices for mechanical dyssynchrony

**Table 4:**
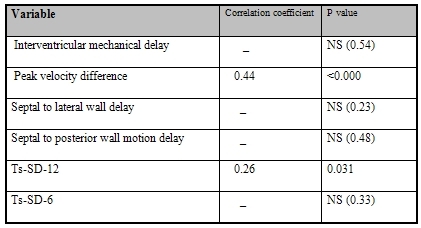
Correlation of low amplitude signal duration and echo indices for mechanical dyssynchrony
